# Genomic and morphological features of an Amazonian *Bacillus thuringiensis* with mosquito larvicidal activity

**DOI:** 10.1186/s13568-025-01850-4

**Published:** 2025-03-05

**Authors:** Veranilce Alves Muniz, Ricardo de Melo Katak, Lílian Caesar, Juan Campos de Oliveira, Elerson Matos Rocha, Marta Rodrigues de Oliveira, Gilvan Ferreira da Silva, Rosemary Aparecida Roque, Osvaldo Marinotti, Olle Terenius, Edmar Vaz de Andrade

**Affiliations:** 1 Graduate Program in Biotechnology - PPGBIOTEC, UFAM, Manaus, Amazonas Brazil; 2 National Institute of Amazonian Research-INPA, Manaus, Amazonas Brazil; 3 Oswaldo Cruz Foundation – Leônidas and Maria Deane Institute, Manaus, Amazonas Brazil; 4 Department of Entomology and Acarology, Luiz de Queiroz College of Agriculture, University of São Paulo, ESALQ – USP - Piracicaba, São Paulo, Brazil; 5 Institute of Biological Sciences - ICB/UFAM, Manaus, Amazonas Brazil; 6 Embrapa Amazônia Ocidental , Manaus, Amazonas Brazil; 7 Department of Biology, Indiana University, Bloomington, IN 47405 USA; 8 Department of Cell and Molecular Biology, Uppsala University, P.O. Box 596, 751 24 Uppsala, Sweden

**Keywords:** *Bacillus* spp., Bioinsecticides, Vector control, Mosquito

## Abstract

The occurrence of mosquito-borne diseases is increasing, and their geographical range is expanding due to climate change. New control measures are urgently needed to combat these debilitating and, in some cases, fatal diseases. Bacteria of the genus *Bacillus* are of interest due to the production of bioactive compounds, including those useful for insect control. The discovery and characterization of new species of *Bacillus* with mosquito larvicidal activity may offer opportunities to develop new products for vector control. In this study, we evaluated larvicidal activity, described morphological characteristics, and sequenced and analyzed the genome of a bacterial strain (GD02.13) isolated from the Amazon region. The metabolites produced by GD02.13 are as effective in killing *Aedes aegypti* larvae as the commercial product Natular™ DT (Spinosad). Furthermore, the morphological characteristics of the GD02.13 spores and crystal inclusions resemble those previously described for *B. thuringiensis*. A phylogenetic analysis based on 443 single-copy orthologs indicated that the bacterial strain GD02.13 belongs to the *Bacillus thuringiensis* species. Its genome, which was assembled and has a size of 6.6 Mb, contains 16 secondary metabolite biosynthetic gene clusters and genes encoding insecticidal proteins, predicted based on sequence similarity. The data obtained in this study support the development of new insecticide products based on the strain GD02.13 of *B. thuringiensis*.

**Supplementary Information:**

The online version contains supplementary material available at 10.1186/s13568-025-01850-4.

## Introduction

*Aedes aegypti* is the main vector of dengue, chikungunya, Zika, and urban yellow fever viruses, which infect thousands of people worldwide (Souza-Neto et al. 2019; Semenza et al. 2022; CDC 2016). Vector control measures targeting *A. aegypti* and other mosquitoes are carried out primarily using chemical insecticides. However, prolonged use of these chemicals harms the environment, human health, and nontarget organisms (Sene et al. 2021). Furthermore, the rapid spread of insecticide resistance highlights the difficulties in controlling vectors worldwide (Demirak and Canpolat 2022). Thus, the quest for sustainable and environmentally friendly alternatives to chemical control of mosquitoes offered numerous innovations (Weng et al. 2023; Aldridge et al. 2024).

Microorganisms have significant potential to control mosquito populations and reduce vector competence, making them alternatives to chemical insecticides (Katak et al. 2023). Bacteria from the Bacillaceae family are known to produce toxins with insecticidal properties and larvicidal activity against several species of mosquitoes (Margalith and Ben-Dov 2000; Boyce et al. 2013; Santana-Martinez et al. 2019). The genus *Bacillus* is a very diverse, evolutionarily and phylogenetically heterogeneous group, including Gram-positive and negative microorganisms, endospore-forming, rod-shaped, aerobic, or facultative anaerobic species, affiliated with the Firmicutes phylum (Logan et al. 2009; Parija 2023). In particular, the *Bacillus thuringiensis* species is relevant for the biological control of mosquitoes that transmit human diseases (Pardo-López et al. 2013; Crickmore et al. 2021; Sánchez–Yáñez et al. 2022; Gangmei et al. 2024; Shikov et al. 2024). Investigation of Bt mechanisms of action led to the discovery of numerous insecticidal molecules (Chaabouni et al. 2012; Salazar et al. 2023; Shikov et al. 2024), and Bt preparations are considered environmentally safe and reliable because they target and eliminate harmful insects without harming non-target animals and plants (Li et al. 2024). For these reasons, commercially available larvicide formulations including strains of Bt are endorsed by organizations such as the World Health Organization (WHO, 2023) and the Environmental Protection Agency (EPA) in the United States of America (www.epa.gov/mosquitocontrol/bti-mosquito-control) (accessed on 12 April 2023).

The discovery of additional microorganisms with mosquitocidal and larvicidal activities, and the characterization of their active metabolites and mechanisms of action is desirable, as they may offer alternative, environmentally friendly insecticides (Vasanthakumari 2019; Ahsan and Shimizu 2021; Milugo et al. 2021; Edmond et al. 2021; Das and Deobhankar 2022; Teshome et al. 2023). The use of native bacterial strains as biological control agents can offer advantages to the management of mosquito populations. These native bacteria are more likely to thrive in local environmental conditions, resulting in more effective and long-lasting control. Furthermore, the genetic diversity of local strains can provide a variety of toxins and mechanisms of action, which helps delay the emergence of resistance in insects. The isolation, characterization, formulation, and finally, field evaluation of local isolates possibly contributes to cost reduction and promotes more sustainable and efficient solutions in biological control (Bravo et al. 2007; Fayad et al. 2019; Brühl et al. 2020).

The Amazon rainforest is an important reservoir of biodiversity on Earth, including fauna, flora, and microorganisms (Heckenberger 2007; Venturini et al. 2022). Bacterial strains previously isolated from Amazon environments showed larvicidal activity against *A. aegypti* (Katak et al. 2021; De Oliveira et al. 2024). In the present study, we further characterized one of these strains, GD02.13, providing additional evaluation of its larvicidal activity, a morphological description, and whole genome sequencing and analysis.

## Methods

### Bacterial strains

The bacterial strain GD02.13 was previously isolated from lake water samples collected in the Municipality of Coari, Amazonas State, Brazil (4°06′45.5″ S 63°07′44.0″ W) (Katak et al. 2021), with the official permission (21263-1) granted by the Biodiversity Authorization and Information System (SISBIO) of the Brazilian Ministry of Environment (MMA). Frozen stocks are kept at the Malaria and Dengue Laboratory (National Institute of Amazonian Research - INPA). The AM65-52 strain of *Bacillus thuringiensis israelensis* (Bti) was obtained from the reactivation of the commercial product Vectobac^®^ WG and used as a reference strain.

### Bioassays with fractionated metabolites

GD02.13 was reactivated from frozen stocks in Luria Bertani (LB) agar plates. An isolated colony was inoculated in 2 ml of LB broth and incubated at 30 °C, 180 rpm for 24 h. Then 50 µL of the culture was transferred to an Erlenmeyer flask containing 100 ml of LB broth and maintained under the same conditions. After 120 h of incubation, cultures were centrifuged for 20 min at 5000 g and the metabolites contained in the supernatant were extracted by a liquid-liquid partition with an equal volume of a mixture of 20% ethyl acetate (AcOEt) and isopropanol (iPr-OH) 9:1 volume/volume (v/v). The extraction was repeated three times, and all the extracted material was combined and dried in a rotary evaporator under vacuum at 45 °C. The recovered, dried sample (supernatant metabolites) was weighed and stored in a desiccator with activated silica (De Oliveira et al. 2021).

Bioassays followed the criteria established by WHO (2005) and Dulmage et al. (1990) and were conducted under controlled conditions of temperature, humidity, and photoperiod. Bioassays were carried out in triplicate, in 150 ml plastic flasks containing 10 ml of distilled water, 10 third instar larvae, larval food (powdered Teklad Global 18% Rodent Diet^®^ pellets), and concentrations of dried supernatant metabolites ranging from 125 to 2.5 µg/ml. All metabolites were solubilized in dimethyl sulfoxide (DMSO; Thermo Fischer Scientific), and mortality readings were recorded at 24, 48, and 72 h after exposure to bacterial extracts (Danga et al. 2014). The DMSO solvent was used as a negative control and Natular™ DT (Spinosad) was the positive control. No mortality was observed when larvae were exposed only to DMSO. The lethal concentration (LC_50_ and LC_90_) was calculated using Probit, with *p* ≤ 0.05 (Finney 1952), and Polo Plus 1.0 statistical software (LeOra Software, Berkeley, CA, USA) (Robertson et al. 2017). Lethal concentrations and confidence interval (95% CI) were analyzed using the Lilliefors normality test (K samples), Tukey’s multiple comparison tests (*p* ≤ 0.05), and Student’s t-test. The BioEstat 5.3 software for Windows was used for the statistical analysis (Ayres et al. 2007).

### Scanning electron microscopy

The GD02.13 and Bti strains were reactivated in solid nutrient agar culture plates (NA = beef extract 1 g, yeast extract 2 g, peptone 5 g, Agar 15 g, per liter, pH 6.8), at 30 °C for 24 h. Isolated colonies were inoculated in nutrient broth (NA minus agar) and incubated for five days at 30 °C in a rotary oven at 180 rpm. The cultures were then centrifuged at 6000 rpm, 4 °C for 20 min. The pellets were washed twice by resuspending in 1 M NaCl, 0.01% Triton X-100 solution followed by centrifugation, and the final pellets were lyophilized. Each lyophilized pellet was suspended in 1 ml of 0.15% NaCl and hexane was added at a rate less than or equal to 10% (50, 75, or 100 µl/ml of aqueous suspension) to minimize the risk of altering the crystals. The suspension was sonicated at 100 W for 10 min to dissipate agglomeration and then centrifuged at 6000 rpm for 10 min. The obtained pellet was resuspended in saline solution, the organic solvent was added again, and the same procedure was repeated four times. Lastly, the visible pellet was washed twice with cold distilled water. This procedure was carried out according to (Loutfi et al. 2020). A scanning electron microscope (JSM-IT500HR), at the Multiuser Center for the Analysis of Biomedical Phenomena at the State University of Amazonas (CMABio), was used to examine and photograph crystals and spores. The morphologies of GD02.13 crystals and spores were analyzed and compared with those of the reference strain AM65-52 (VectoBac WG) (Mounsef et al. 2014).

### Genome sequencing, assembly and statistics

Genomic DNA was extracted using the DNeasy PowerSoil Pro Kits – QIAGEN and sequencing was performed on an Illumina MiSeq instrument using the 2 × 150 bp paired-ends protocol. The “de novo” assembly was performed using SPAdes v.3.14.1 (Prjibelski et al. 2020), including the flag -*-careful* to reduce the number of mismatches and short indels in the assembly. CheckM v.1.1.6 (Parks et al. 2015) was run as default to retrieve information on genome completeness and contamination. Reads were mapped against all contigs with Bowtie2 v.2.4.2 (Langmead and Salzberg 2012) using the flag *--no-discordant* to allow only alignments where both mates align equally, and the output was used with Samtools v.1.15.1 (Danecek et al. 2021) to recover genome average coverage.

### Genome annotation and phylogeny

The protein coding sequences present in the GD02.13 genome were predicted and annotated using the default parameters of Prokka v.1.14.6 (Seemann 2014). The 16S rRNA sequence was used as a query in BLASTn (Camacho et al. 2009) against the NCBI nt database (Sayers et al. 2022) to identify broad strain taxonomy and guide the choice of reference genomes to include in the phylogenetic analyses. OrthoFinder v.2.5.4 (Emms and Kelly 2019) was used as default to recover single-copy orthologs among all genomes, and IQ-TREE2 v.2.2.0.3 (Minh et al. 2020) was used to run the model test and infer phylogenetic trees by maximum likelihood.

### Prediction of biosynthetic gene clusters and insecticidal genes

The biosynthetic gene clusters were predicted from the strain genome with AntiSMASH v.7 (Blin et al. 2023) using the default website software. The identified clusters were searched against the MIBiG repository (Medema et al. 2015). To determine whether BGCs were integrated into the main chromosome, plasmid, or integrated bacteriophages, these two types of mobile genetic elements were predicted with GeNomad v.1.4.0 (Camargo et al. 2023) using the default command. The genes encoding insecticidal toxins were mined with BtiToxin_Digger v1.0.10 (Liu et al. 2022), with the target insect species derived from the BPPRC specificity database (https://www.bpprc-db.org/, accessed on 21 March 2024) (Panneerselvam et al. 2022).

The sequencing reads used in the analysis, corresponding to the *Bacillus thuringiensis*-GD02.13 strain, have been deposited in the National Center for Biotechnology Information (NCBI) database (https://www.ncbi.nlm.nih.gov/) as BioProject PRJNA1137396, Accession numbers SAMN42590146.

## Results

### Larvicidal activity of GD02.13 metabolites

Previously, we demonstrated that the bacterial strain GD02.13 is lethal to *A. aegypti* larvae in concentrations comparable to those observed with the Bti strain AM65-52. Now, we demonstrate that metabolites extracted from GD02.13 are effective larvicides at concentrations equivalent to those of the commercial product Natular™ DT. While the LC_50_ and LC_90_ determined for GD02.13 extract and Natular™ DT were similar at 24h of exposure, the LC_90_ values of the GD02.13 extracts and Natular™ DT were statistically different (*p* ≤ 0.05) in the 48- and 72-hour intervals, with the GD02.13 extract being more efficient. Because of the high larval mortality observed after 48–72 h of exposure to Natular™ DT or GD02.13 extract (> 50%), at the concentrations applied in our assays, LC_50_ values could not be estimated with our experimental protocol.

**Table 1:** LC_50_ and LC_90_ values of bacterial metabolites against *A. aegypti* larvae

Interval	Strain	LC_50_ µg/ml (CI 95%)	χ^2^	df	Slope ± SE	LC_90_ µg/ml (CI 95%)	χ^2^	df	Slope ± SE
24 h	Spinosad	4.6 (3.4–5.7)^a^	8.9	7	1.8 ± 0.14	23.3 (18.2–32.6)^b^	8.9	7	1.8 ± 0.14
	GD02.13	3.2 (2.3–4.1)^a^	7.8	7	1.8 ± 0.17	15.6 (12.3–21.3)^b^	7.8	7	1.9 ± 0.17
48 h	Spinosad	-	-	-	-	13.9 (11.5–17.6)^c^	5.4	7	1.8 ± 0.17
	GD02.13	-	-	-	-	5.0 (4.1–6.3)^d^	3.5	7	2.2 ± 0.34
72 h	Spinosad	-	-	-	-	6.1 (5.0–7.8)^e^	3.2	7	1.9 ± 0.28
	GD02.13	-	-	-	-	2.3 (1.0–3.1)^f^	1.3	7	2.3 ± 0.72

Values expressed in micrograms of extracts per 72 h per assay. Dead larvae were counted 24, 48, and 72 hours after exposure to the extracts. LC_50_ and LC_90_ were evaluated by Probit, with *p* ≤0.05. Statistical comparisons and confidence intervals (95 % CI) were analyzed using the Lilliefors normality test (K samples), Tukey’s multiple comparison test (*p* ≤0.05), and Student t-test. Student's t. For all variables in each column with the same letter (a.b.c.d.e.f), the differences between the values are not statistically significant LC = lethal concentration; CI = confidence interval; *x*^*2*^ = chi-square; df = degrees of freedom; Natular™ DT positive control

### Morphological characterization

**Fig. 1:**
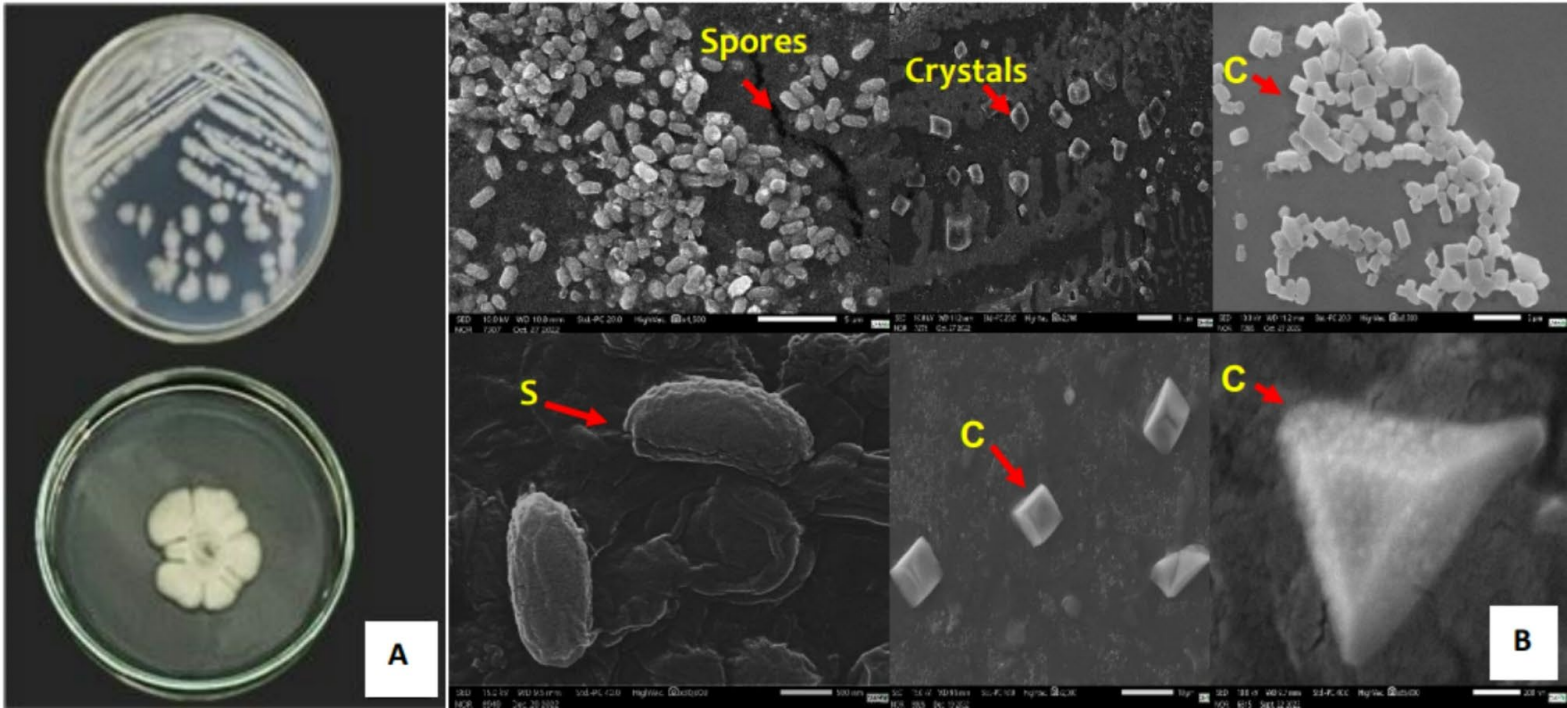
GD02.13 morphology **A.** Colony morphology of strain GD02.13 after one day of cultivation on Nutrient Agar medium **B.** Scanning electron micrograph of GD02.13 spores and crystals. Spores (S) and protein crystals (C)

The GD02.13 lineage grows in Nutrient Agar medium, forming flat, opaque colonies that are whitish-gray I color, rounded, and with wavy margins. Scanning electron microscopy was performed to investigate the occurrence of crystalline inclusions and spores in GD02.13 bacteria. The images show that the GD02.13 strain contains inclusions and produces spores resembling those of *Bacillus thuringiensis*, with crystalline forms in cuboidal, spherical, and undefined shapes (Fig. 1).

### Genome sequencing and phylogenetic analysis

Sequencing of the GD02.13 genome yielded 7,674,646 paired-end reads, which were assembled into 65 contigs > 2000 bp (Table 2). Smaller contigs were removed, as they likely represent assembly noise resulting from using short reads or due to complex genome regions. The assembly quality check also confirmed that the removal of small contigs does not affect the completeness of the genome and, in fact, improves cleanliness by eliminating misassembled contigs with extra single-copy genes (Table S2).

**Table 2:** Features of the genome of *Bacillus thuringiensis* GD02.13

Species	Accession	Size (Mb)	GC%	N50	Coverage	Contigs	Completeness	CDS
*B. thuringiensis*	SAMN42590146	6.6	34.83	274.165	339.4	65	99.43	7367

**Fig. 2:**
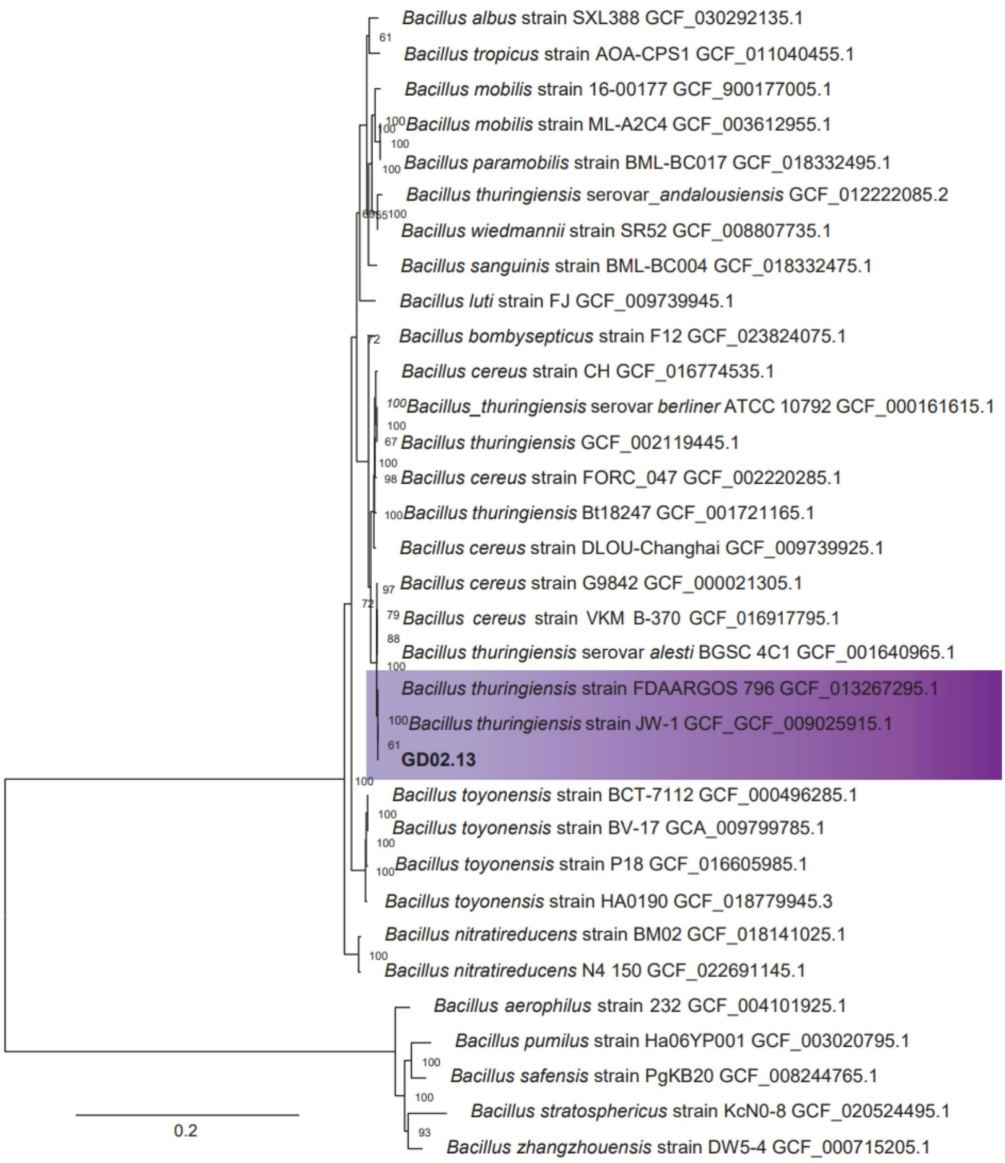
Phylogenetic inference of GD02.13 based on the whole genome by maximum likelihood

Phylogenetic inference based on 443 single-copy orthologs (bootstrap 1000, LG + F + G4 model) indicated that GD02.13 is a strain of *Bacillus thuringiensis* (Fig. 2) that clusters with two previously isolated *B. thuringiensis*, one from China (strain JW-1, GCF_009025915. 1) and another from the USA (strain FDAARGOS_796, GCF_013267295.1).

### Analysis of biosynthetic gene clusters and insecticidal genes

**Fig. 3:**
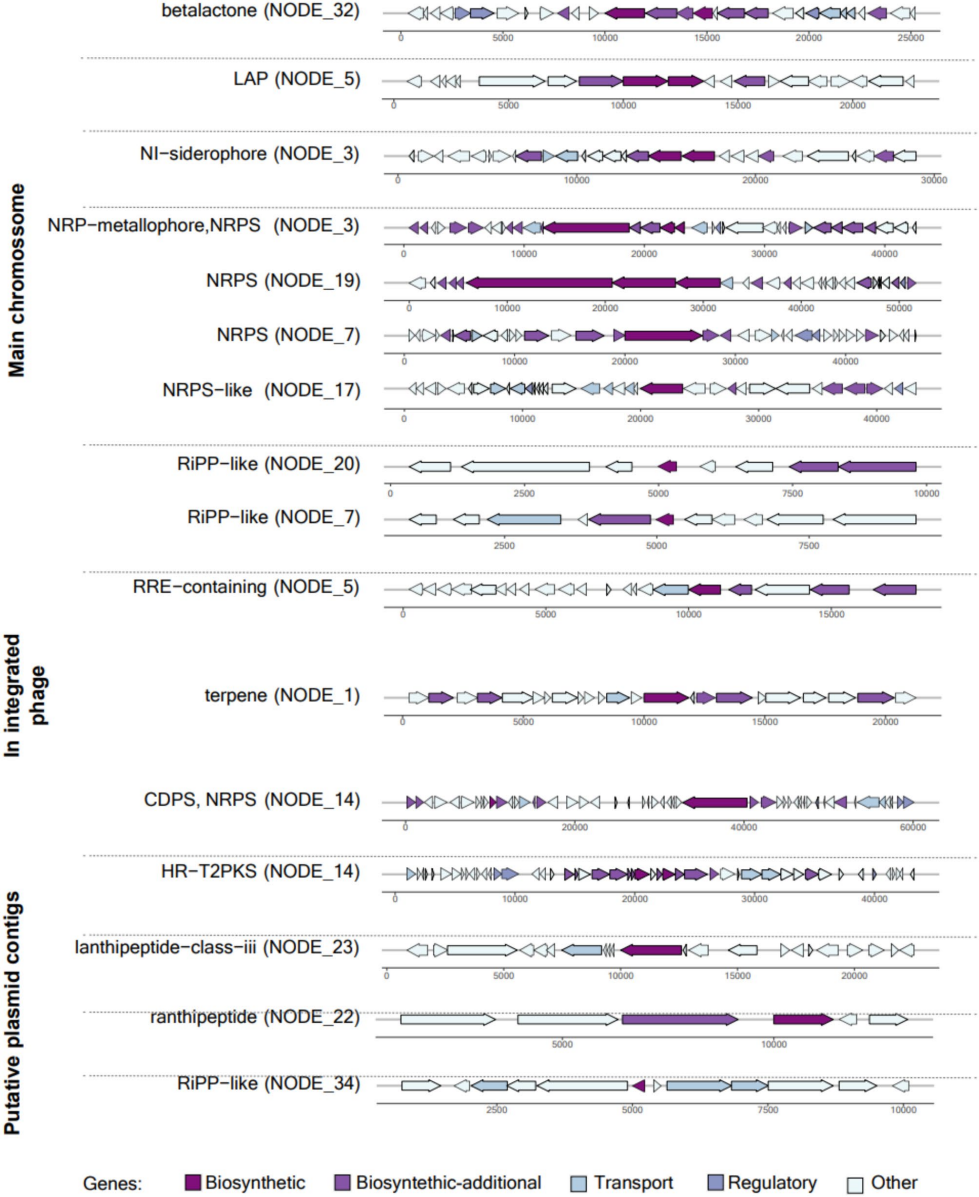
Prediction of biosynthetic gene clusters (BGCs) in a *Bacillus thuringiensis* GD02.13 strain with the antiSMASH database and the MIBIG repository

A total of 16 biosynthetic gene clusters (BGC) were predicted in the GD02.13 genome (Fig. 3). Eleven BGCs are in the contigs comprising the main chromosome, while one terpene biosynthetic gene cluster is part of an integrated Caudoviricetes phage genome. Five BGCs, CDPNRPS, HRT2PKS, lanthipeptide class iii, ranthipeptide, and RiPPlike, were predicted to be in putative plasmids. It is worth mentioning that the petrobactin BGCs exhibited 100% similarity to the known entity, while bacillibactin, pulchenimic, fengycin, and cerecyclin varied between 85% and 30% similarity to their corresponding gene clusters (Table S1).

The genome encodes several proteins with high similarities with previously characterized *B. thuringiensis* insecticidal toxins, specifically Cry11Aa3, Cry6Ba3, Cry60Ba3, Cry6Aa3, CryBa, Cry4Ba, Cry4Ba2, Cry4ba4, Cryba2, Cry4Ba4 and Cyt (Table S3).

## Discussion

In our previous work (Katak et al. 2021) we isolated Amazonian-native environmental bacterial strains with larvicidal activities against *(A) aegypti.* One of those, named GD02.13, is as active in killing mosquito larvae as is the *(B) thuringiensis* strain AM65-52 (Vectobac WG) (Katak et al. 2021). To further assess the potential of GD02.13 for mosquito control applications, we compared the lethality of its metabolites with the commercial insecticide Natular™ DT, a product obtained from aerobic fermentation of *Sacharopolyspora spinosa*, and composed of a mixture of the metabolites, spinosad A and D (Fernandes et al. 2019; Vicari et al. 2023). The GD02.13 metabolites are lethal to *(A) aegypti* larvae, in concentrations equivalent to those of Natular™ DT. Furthermore, our study showed, through scanning electron microscopy, that GD02.13 produces crystals and spores (Fig. 1) with morphologies that resemble those of *(B) thuringiensis* strain AM65-52 (Vectobac^®^ WG). The observed GD02.13 spores and various sizes and shapes of crystals are consistent with previous morphological descriptions of *Bacillus* strains (Fayad et al. 2019; Xie et al. 2019; Loutfi et al. 2020). These results support additional efforts to characterize GD02.13, its insecticidal metabolites and proteins, and their mechanisms of action.

Whole genome sequencing, analyses, and mining are powerful in establishing taxonomic assignment for bacteria belonging to species complexes, uncovering potential insecticidal candidates, understanding the genetic basis of their production, and discovering new active metabolites (Meesil et al. 2023; Albarano et al. 2020).

Accordingly, in the present study, we applied a genome-based multigene phylogeny approach that improves phylogenetic relationship assignments (Jeong Haeyoung et al. 2017; Lechuga et al. 2020; Patel et al. 2020). *B. thuringiensis* belongs to the *Bacillus cereus* complex or *B. cereus* s. l., in which taxonomic inconsistencies frequently cause problems with accurate species identification (Lazarte et al. 2018; Ehling-Schulz et al. 2019). According to our data and phylogenetic analysis, GD02.13 groups with *B. thuringiensis* strains JW1 (GCF_009025915.1) and FDAARGOS_796 (GCF_013267295.1) (Fig. 2), indicating that GD02.13 is a new strain of *B. thuringiensis.*

It is well established that *B. thuringiensis* produces secondary metabolites with larvicidal activity against several insects, including medically important mosquito species (Dahmana et al. 2020; Falqueto et al. 2021; Sujayanand et al. 2023). These metabolites are frequently synthesized by genes arranged in clusters (Biosynthetic Gene Clusters - BGCs). Relevantly, we identified several BCGs in the genome of GD02.13. Among them, BGCs associated with the synthesis of Lantipeptides, Rantipeptides, ribosomally synthesized and post-translationally modified peptides (RiPPs), type II polyketides (T2PKS) (Fig. 3), Petrobactin, molybdenum cofactor and Fengycin (Table S1) were previously described by Shikov et al. (2024) to be present in the genome of the *B. thuringiensis* highly insecticidal strain 800/15. One terpene biosynthetic gene cluster is within the sequence of an integrated Caudoviricetes phage. Caudovirales, have been identified in many species of the *B. cereus* group (Gillis and Mahillon 2014).

In addition to the identified BCGs, GD02.13 contains genes coding for insecticidal proteins. In general, genes coding for insecticidal proteins are located on plasmids, as revealed in the sequenced and assembled GD02.13 genome and GD02.13 plasmids. The genes identified in this study code for a series of insecticidal proteins, including well-characterized Cry and Cyt toxins that are active against numerous insects of the order Diptera, including *Aedes*,* Anopheles*, and *Culex* species, vectors of human diseases. These proteins accumulate during sporulation in large parasporal crystals that consist mainly of Cry proteins and, in some strains, cytotoxic Cyt proteins (Fayad et al. 2019; Xie et al. 2019; Loutfi et al. 2020).

In summary, we added information about the larvicidal properties of GD02.13 metabolites, by comparing its activity with the commercial product Natular™ DT. Whole genome sequencing and analysis revealed several potential insecticidal candidates including secondary metabolites and proteins. Isolation and characterization of these potential active molecules will further inform about the mechanisms by which GD02.13 kills mosquito larvae. Assays on medium and large scales under natural conditions will be necessary to evaluate GD02.13 performance and stability and advise about formulations that may be applied for mosquito control. GD02.13 and other larvicidal native bacterial strains are expected to facilitate area-wide control or elimination of *Ae. aegypti* if properly developed and adopted as part of an integrated pest management strategy.

## Electronic supplementary material

Below is the link to the electronic supplementary material.

Supplementary Material 1

## Data Availability

All data generated or analyzed during this study are included in this published article.

## Abbreviations

SISBIO: Biodiversity Authorization and Information System
MMA: Brazilian Ministry of Environment
Bti: *Bacillus thuringiensis israelensis*
LB: Luria Bertani
AcOEt: Ethyl acetate
iPr-OH: Isopropanol
DMSO: Dimethyl sulfoxide
NA: Nutrient agar
NCBI: National Center for Biotechnology Information
BGC: Biosynthetic gene clusters
SIT: Sterile insect technique
IIT: Incompatible insect technique
Lbs: *Lysinibacillus sphaericus*
EPA: Environmental Protection Agency
