# Supplementary material for: Genomic and morphological features of an Amazonian Bacillus thuringiensis with mosquito larvicidal activity
Source: AMB Express. 2025 Mar 5;15:39. doi: 10.1186/s13568-025-01850-4 (PMC11882490; doi:10.1186/s13568-025-01850-4)
Supplement: Supplementary file 1 — Supplementary Material 1 [file 13568_2025_1850_MOESM1_ESM.docx]

**Supplementary material**

Table S1: Biosynthetic gene clusters (BGC) predicted in the *B. thuringiensis* GD02.13 genome

| Contig | | Tool | Type/Activity | | | | Location (relative coordinate, b. p.) | | Most Similar Known Cluster | | Similarity | Score |
| --- | --- | --- | --- | --- | --- | --- | --- | --- | --- | --- | --- | --- |
| 1 | antiSMASH | | | Terpene | | 177,786-199,639 (21,854) | | Molybdenum cofactor | | | 17% | 0.27 |
| 3.1 | antiSMASH | | | NRP-metallofore - NRPS | 1-43,342 (43,342) | | | bacilicactin | | | 85% | 1.11 |
| 3.2 | antiSMASH | | | NI – siderophore | 330,830–360,370 (29,541) | | | [petrobactin](https://mibig.secondarymetabolites.org/go/BGC0000942/1) | | | 100% | 0.69 |
| 5.1 | antiSMASH | | | LAP | 2,808 - 26,314 (23,507) | | | - | | - | | 0.26 |
| 5.2 | antiSMASH | | | [RRE-containing](https://docs.antismash.secondarymetabolites.org/glossary/#rre-containing) | 338,791 - 356,760 (17,970) | | | - | | - | | 0.17 |
| 7.1 | antiSMASH | | | NRPS | 179,186 - 226,202 (47,017) | | | - | | - | | 0.31 |
| 7.2 | antiSMASH | | | [RiPP-like](https://docs.antismash.secondarymetabolites.org/glossary/#ripp-like) | 244,898 - 255,158 (10,261) | | | - | | - | | - |
| 14.1 | antiSMASH | | | HR – T2PKS | 3,213 - 46,585 (43,373) | | | - | | - | | 0.32 |
| 14.2 | antiSMASH | | | [CDPS, NRPS](https://docs.antismash.secondarymetabolites.org/glossary/#nrps) | 67,311 - 127,676 (60,366) | | | [pulcherriminic acid](https://mibig.secondarymetabolites.org/go/BGC0002103/1) | | 66% | | 0.58 |
| 17.1 | antiSMASH | | | [NRPS-like](https://docs.antismash.secondarymetabolites.org/glossary/#nrps-like) | 77,557 - 121,138 (43,582) | | | - | | - | | 0.30 |
| 19.1 | antiSMASH | | | [NRPS](https://docs.antismash.secondarymetabolites.org/glossary/#nrps) | 1 - 51,734 (51,734) | | | - | | - | | 0.45 |
| 20.1 | antiSMASH | | | [RiPP-like](https://docs.antismash.secondarymetabolites.org/glossary/#ripp-like) | 59,796 - 70,125 (10,330) | | | - | | - | | 0.08 |
| 22.1 | antiSMASH | | | [ranthipeptide](https://docs.antismash.secondarymetabolites.org/glossary/#ranthipeptide) | 84,574 - 97,838 (13,265) | | | - | | - | | 0.20 |
| 23.1 | antiSMASH | | | [lanthipeptide-class-iii](https://docs.antismash.secondarymetabolites.org/glossary/#lanthipeptide-class-iii) | 1,124 - 23,727 (22,604) | | | - | | - | | 0.27 |
| 32.1 | antiSMASH | | | [betalactone](https://docs.antismash.secondarymetabolites.org/glossary/#betalactone) | 10,424 - 35,662 (25,239) | | | [fengycin](https://mibig.secondarymetabolites.org/go/BGC0001095/1) | | 40% | | 0.26 |
| 34.1 | antiSMASH | | | [RiPP-like](https://docs.antismash.secondarymetabolites.org/glossary/#ripp-like) | 2,930 - 13,151 (10,222) | | | [cerecyclin](https://mibig.secondarymetabolites.org/go/BGC0002447/1) | | 30% | | 0.32 |
|  | | |  | |  | | |  | |  | |  |

Table S2

| **Genome** | **Id** | **Marker_lineage** | | **#genomes** | **#markers** | **#marker_sets** | **0** | **1** | | **2** | **3** |  | **5+** | **Completeness** | **Contamination** | **Strain_**  **heterogeneity** | |  |  |
| --- | --- | --- | --- | --- | --- | --- | --- | --- | --- | --- | --- | --- | --- | --- | --- | --- | --- | --- | --- |
| contigs5000l | o__Bacillales | (UID828) | 139 | | 508 | 174 | 1 | 496 | | 9 | 2 | 0 | 0 | 99,43 | 2.36 | 0 |  |  |  |
| **contigs2000l** | **o__Bacillales** | **(UID828)** | **139** | | **508** | **174** | **1** | **496** | | **9** | **2** | **0** | **0** | **99,43** | **2.36** | **0** |  |  |  |
| contigs500l | o__Bacillales | (UID828) | 139 | | 508 | 174 | 1 | 457 | 46 | | 4 | 0 | 0 | 99,43 | 6.89 | 7 |  | |  |
| contigsALL | k__Bacteria | (UID203) | 5449 | | 101 | 56 | 1 | 47 | 35 | | 14 | 3 | 1 | 98,21 | 52,1 | 7 |  | | |

| Proteins type | Protein ID | Protein description | length | Best-hit | Hit length | coverage | Identity | target order | Target species |
| --- | --- | --- | --- | --- | --- | --- | --- | --- | --- |
| Proteins cry | [fig\|1428.2295.peg.6643](https://bcam.hzau.edu.cn/cgi-bin/BtToxin_scanner/sequence.php?ID=fig\|1428.2295.peg.6643&filename=../../BtToxin_scanner/download/f7671b5c027c266ad39d2e928281df59.faa) | Protein hypothetical 1428.2295 | 643 | Cry11Aa3 | 643 | 100.00 | 100 | Diptera | *Culex quinquefasciatus*, *Anopheles albimanus* e *Aedes aegypti* |
| Proteins cry | [fig\|1428.2295.peg.6764](https://bcam.hzau.edu.cn/cgi-bin/BtToxin_scanner/sequence.php?ID=fig\|1428.2295.peg.6764&filename=../../BtToxin_scanner/download/f7671b5c027c266ad39d2e928281df59.faa) | Protein hypothetical\| 1428.2295] | 319 | Cry60Ba3 | 319 | 100.00 | 100.00 |  | ND |
| Proteins Cry | [fig\|1428.2295.peg.6765](https://bcam.hzau.edu.cn/cgi-bin/BtToxin_scanner/sequence.php?ID=fig\|1428.2295.peg.6765&filename=../../BtToxin_scanner/download/f7671b5c027c266ad39d2e928281df59.faa) | Protein hypothetical 1428.2295] | 303 | Cry60Aa3 | 303 | 100.00 | 100.00 |  |  |
| Proteins cry | [fig\|1428.2295.peg.6809](https://bcam.hzau.edu.cn/cgi-bin/BtToxin_scanner/sequence.php?ID=fig\|1428.2295.peg.6809&filename=../../BtToxin_scanner/download/f7671b5c027c266ad39d2e928281df59.faa) | Protein hypothetical 1428.2295] | 918 | Cry4Ba | 1136 | 80.81 | 100.00 | Diptera | *Aedes aegypti, Culex quinquefasciatus, Anopheles gambie, Anopheles albimanus e Anopheles stephensis.* |
| Proteins Cry | [fig\|1428.2295.peg.6882](https://bcam.hzau.edu.cn/cgi-bin/BtToxin_scanner/sequence.php?ID=fig\|1428.2295.peg.6882&filename=../../BtToxin_scanner/download/f7671b5c027c266ad39d2e928281df59.faa) | hypothetical1428.2295] | 133 | Cry4Ba | 1136 | 11.71 | 69.92 |  |  |
| Proteins Cry | [fig\|1428.2295.peg.6883](https://bcam.hzau.edu.cn/cgi-bin/BtToxin_scanner/sequence.php?ID=fig\|1428.2295.peg.6883&filename=../../BtToxin_scanner/download/fb587fa1683ff4714c1a6696a9867b94.faa) | Protein hypothetical [1428.2295] | 208 | Cry4Ba2 | 1136 | 18.31 | 100.0 | Diptera |  |
| Proteins Cry | [fig\|1428.2295.peg.6974](https://bcam.hzau.edu.cn/cgi-bin/BtToxin_scanner/sequence.php?ID=fig\|1428.2295.peg.6974&filename=../../BtToxin_scanner/download/fb587fa1683ff4714c1a6696a9867b94.faa) | Protein hypothetical  [1428.2295] | 155 | Cry4Ba4 | 1135 | 13.74 | 65.38 | Diptera |  |
| Proteins Cry | [fig\|1428.2295.peg.6960](https://bcam.hzau.edu.cn/cgi-bin/BtToxin_scanner/sequence.php?ID=fig\|1428.2295.peg.6960&filename=../../BtToxin_scanner/download/fb587fa1683ff4714c1a6696a9867b94.faa) | Protein hypothetical 1428.2295] | 208 | Cry4Ba2 | 1136 | 18.31 | 100.00 | Diptera |  |
| Proteins Cry | [fig\|1428.2295.peg.6974](https://bcam.hzau.edu.cn/cgi-bin/BtToxin_scanner/sequence.php?ID=fig\|1428.2295.peg.6974&filename=../../BtToxin_scanner/download/fb587fa1683ff4714c1a6696a9867b94.faa) | Protein hypothetical  1428.2295 | 155 | Cry4Ba4 | 1135 | 13.74 | 65.38 | Diptera |  |
| Proteins Cyt | [fig\|1428.2295.peg.5841](https://bcam.hzau.edu.cn/cgi-bin/BtToxin_scanner/sequence.php?ID=fig\|1428.2295.peg.5841&filename=../../BtToxin_scanner/download/fb587fa1683ff4714c1a6696a9867b94.faa) | PROTEÍNA DA TOXINA MOSQUITOCIDA 1428.2295 | 515 | ND | ND | ND | ND |  |  |
| Proteins Cyt | [fig\|1428.2295.peg.6641](https://bcam.hzau.edu.cn/cgi-bin/BtToxin_scanner/sequence.php?ID=fig\|1428.2295.peg.6641&filename=../../BtToxin_scanner/download/fb587fa1683ff4714c1a6696a9867b94.faa) | Protein hipotética 1428.2295 | 249 | ND | ND | ND | ND |  |  |
| Proteins Cyt | [fig\|1428.2295.peg.6807](https://bcam.hzau.edu.cn/cgi-bin/BtToxin_scanner/sequence.php?ID=fig\|1428.2295.peg.6807&filename=../../BtToxin_scanner/download/fb587fa1683ff4714c1a6696a9867b94.faa) | protein hypothetical 1428.2295 | 525 | ND | ND | ND | ND |  |  |
| Proteins Cyt | [fig\|1428.2295.peg.6810](https://bcam.hzau.edu.cn/cgi-bin/BtToxin_scanner/sequence.php?ID=fig\|1428.2295.peg.6810&filename=../../BtToxin_scanner/download/fb587fa1683ff4714c1a6696a9867b94.faa) | Delta-endotoxina cytolytic type 1Ba 1428.2295 | 263 | ND | ND | ND | ND |  |  |
| Proteins Cyt | [fig\|1428.2295.peg.6912](https://bcam.hzau.edu.cn/cgi-bin/BtToxin_scanner/sequence.php?ID=fig\|1428.2295.peg.6912&filename=../../BtToxin_scanner/download/fb587fa1683ff4714c1a6696a9867b94.faa) | proteína hipotética 1428.2295 | 471 | ND | ND | ND | ND |  |  |

Table S3: Analysis of genes encoding insecticidal toxins mined with BtiToxin_Digger v1.0.10 using target insect species derived from the BPPRC specificity database
